# Predictive capacity of a genetic risk score for coronary artery disease in assessing recurrences and cardiovascular mortality among patients with myocardial infarction

**DOI:** 10.3389/fcvm.2023.1254066

**Published:** 2023-09-14

**Authors:** Luis Miguel Rincón, Isaac Subirana, Candelas Pérez del Villar, Pedro L. Sánchez, José Luis Zamorano, Jaume Marrugat, Roberto Elosua

**Affiliations:** ^1^ Cardiology Department, Hospital Universitario de Salamanca–IBSAL, Universidad de Salamanca, Salamanca, Spain; ^2^ Centro de Investigación Biomédica en Red de Enfermedades Cardiovasculares (CIBERCV), Madrid, Spain; ^3^ Universidad de Alcalá, Madrid, Spain; ^4^ Hospital del Mar Medical Research Institute, Barcelona, Spain; ^5^ Cardiology Department, Hospital Ramón y Cajal, Universidad de Alcalá, Madrid, Spain; ^6^ Faculty of Medicine, University of Vic-Central University of Catalonia, Vic, Spain

**Keywords:** genetic risk score, myocardial infarction, recurrences, secondary prevention, precision medicine

## Abstract

**Aim:**

This study aimed to evaluate the capacity of a genetic risk score (GRS) for coronary artery disease (CAD) independent of classical cardiovascular risk factors to assess the risk of recurrence in patients with first myocardial infarction. The secondary aim was to determine the predictive value of this GRS.

**Methods:**

We performed a meta-analysis of individual data from three studies, namely, a prospective study including 75 patients aged <55 years, a prospective study including 184 patients with a mean age of 60.5 years, and a case–control study (77 cases and 160 controls) nested in a cohort of patients with first myocardial infarction. A GRS including 12 CAD genetic variants independent of classical cardiovascular risk factors was developed. The outcome was a composite of cardiovascular mortality and recurrent acute coronary syndrome.

**Results:**

The GRS was associated with a higher risk of recurrence [hazard ratio = 1.24; 95% confidence interval (CI): 1.04–1.47]. The inclusion of the GRS in the clinical model did not increase the model’s discriminative capacity (change in C-statistic/area under the curve: 0.009; 95% CI: −0.007 to 0.025) but improved its reclassification (continuous net reclassification index: 0.29; 95% CI: 0.08–0.51).

**Conclusion:**

The GRS for CAD, independent of classical cardiovascular risk factors, was associated with a higher risk of recurrence in patients with first myocardial infarction. The predictive capacity of this GRS identified a subgroup of high-risk patients who could benefit from intensive preventive strategies.

## 1. Introduction

Genome-wide association studies have helped unravel the genetic architecture of coronary artery disease (CAD) and identified more than 150 CAD-related loci (1). Most of these loci are associated with CAD risk factors, but some are independent, pointing to new CAD pathogenic mechanisms. On the one hand, these loci could identify new therapeutic targets to reduce residual cardiovascular risk. On the other hand, several studies have used a genetic risk score (GRS) in predicting and identifying high-risk individuals in primary prevention (2–7). We developed and validated a GRS including 12 genetic variants independent of classical risk factors in several populations in primary prevention (3, 6).

However, the role of GRS in the prognosis of patients with acute coronary syndrome (ACS) has been less explored (8–15). We hypothesized that the GRS, which was previously developed and validated in a primary prevention setting and included CAD genetic variants independent of classical cardiovascular risk factors, could also predict ACS recurrences or even death despite intensive secondary preventive strategies and pharmacological treatments in patients who already have the disease.

This study aimed to assess the predictive capacity of a CAD GRS, independent of classical cardiovascular risk factors, for the risk of recurrence or cardiovascular mortality in patients with first ACS.

## 2. Materials and methods

### 2.1. Design and participants

We performed a meta-analysis of individual data from three studies:

• The first study included 81 consecutive nondiabetic patients aged <55 years who presented with acute myocardial infarction (AMI) in a tertiary hospital (16). This prospective study had a median follow-up duration of 4.1 years. Only 75 patients with first AMI were considered for the present analysis.
• The second study also had a prospective design (17) and included 184 consecutive patients, with a mean age of 60.5 years, who presented with first AMI in the same tertiary hospital. The median follow-up duration was 1.6 years.
• The third study, with a 2-year follow-up duration, was an age- and sex-matched case–control (1:2) study nested in a cohort of consecutive patients who presented with first AMI in another tertiary hospital [viz., REGICOR (Registre Gironi del Cor or Girona Heart Registry) study]. This REGICOR study subsample, with a 2-year clinical follow-up duration, included a hospital myocardial infarction registry (18) comprising 1,141 patients who had donated their DNA.
  In this setting, we designed a nested age- and sex-matched case–control study. The case group included 86 patients who presented with a new ACS event or death during the 2-year follow-up. The control group included 172 age- and sex-matched patients, who were randomly selected among those who did not present such events. Finally, 77 and 160 patients in the case and control groups, respectively, were included due to the quality of the DNA obtained.

### 2.2. Genetic variants, genotyping, and GRS

We analyzed eight genetic variants previously identified to be associated with CAD but not with the classical cardiovascular risk factors (total cholesterol, low-density lipoprotein cholesterol, high-density lipoprotein cholesterol, blood pressure, smoking, or diabetes mellitus), namely, single-nucleotide polymorphisms (SNPs) rs17464857 in *MIA3*, rs6725887 in *WDR12*, rs9818870 in *MRAS*, rs10455872 in *SLC22A3-LPAL2-LPA*, rs12526453 in *PHACTR1*, rs1333049 in *CDKN2B-AS1*, rs501120 in *CXCL12*, and rs9982601 in *KCNE2-SCL5A3*.

We also incorporated four variants of the *ALOX5AP* haplotype B, which has been reported to be associated with CAD in different populations (19–21). This haplotype consisted of rs10507391-A, rs9315050-A, rs17222842-G, and rs17216473-A. A GRS including these 12 genetic variants (eight SNPs and four haplotype variants) was defined as the sum of the number of risk alleles/haplotype across all genetic variants after weighting each one by its estimated effect size in the CARDIoGRAMplusC4D [Coronary Artery Disease Genome-Wide Replication and Meta-Analysis Plus the Coronary Artery Disease (C4D) Genetics] consortium (21). In addition, a weight of 0.131 [odds ratio (OR) = 1.14] was assigned to the *ALOX5AP* haplotype B.

DNA extraction was performed using standard techniques. The 12 genetic variants were determined, and the GRS was calculated using the CARDIO inCode Score commercial platform (GENinCode Plc, Oxford, UK). This GRS had been previously developed and validated in several populations in primary prevention (3, 6). Individual and genotype call rates and Hardy–Weinberg equilibria were also evaluated.

### 2.3. Outcomes

All the participants were followed up every 6 months at the outpatient clinic in the tertiary hospital where the patients for the first two studies were recruited. In the REGICOR Study, follow-up was conducted through data linkage with population-based myocardial infarction records, official mortality registries, and telephone interviews.

The primary outcome, defined as a recurrent cardiovascular event, was a composite of cardiovascular mortality and recurrent ACS, including both AMI and unstable angina. Nonculprit lesions that were revascularized were not considered recurrences. All outcomes were reviewed by two cardiologists in the first two studies and by an event committee in the REGICOR study.

### 2.4. Other covariates

The following variables were also collected: age; sex; maximum Killip class during index hospitalization; family history of CAD; personal history of hypertension, dyslipidemia, diabetes, and smoking; treatment with beta-blockers; angiotensin-converting enzyme inhibitors; and revascularization.

### 2.5. Statistical analysis

The quantitative variables were expressed as means and standard deviations, and the qualitative variables were expressed as counts and percentages. Student’s *t*-test and ANOVA were used to compare the quantitative variables, and the chi-squared test was used to compare the qualitative variables between the two groups.

In the multivariable analyses, Cox survival regression models were used in the first two studies and logistic regression was used in the case–control study. A clinical regression model, including age, sex, maximum Killip class during hospital stay, family history of CAD, and personal history of diabetes, hypertension, dyslipidemia, and smoking, was initially defined. The final model included those factors associated with the outcome in any of the studies. The GRS was added to this widely used clinical model. The added predictive value of the GRS was assessed by the improvement of the discriminative capacity [change in the C-statistic or area under the curve (AUC)] and reclassification (integrated discrimination improvement index and continuous net reclassification improvement).

We used the inverse variance method to weight the results of the three studies and the fixed effects model to estimate the summary effect size of the association and the predictive improvement metrics. We also assessed the heterogeneity of the results across studies.

A *p*-value of <0.05 was considered statistically significant. All the statistical analyses were performed using R version 3.3.2.

## 3. Results

### 3.1. Results of the individual studies

Participant and genotype call rates were >99%, and all the genetic variants analyzed followed the Hardy–Weinberg equilibrium. Table 1 shows the main characteristics of the participants in the three studies. Table 2 shows the differences between patients with and without an event during follow-up. The group of participants with events had a higher Killip class and standardized GRS. The association between a family history of CAD and a higher risk of events was inconsistent across studies, with two studies showing a lower proportion of family history among patients with events and the third showing the opposite. No other significant differences were observed.

**Table 1:** Main characteristics of the participants in the three studies included in this meta-analysis.

	Study 1 (*n* = 75)	Study 2 (*n* = 184)	REGICOR (*n* = 237)
Age (years)	48.0 (6.1)	63.0 (14.1)	61.9 (9.5)
Sex (male), *n* (%)	66 (88.0)	146 (79.3)	162 (68.4)
Smoking, *n* (%)	49 (65.3)	67 (36.4)	92 (39.1)
Hypertension, *n* (%)	28 (37.3)	113 (61.4)	138 (60.3)
Diabetes, *n* (%)	0 (0)	51 (27.7)	69 (31.4)
Dyslipidemia, *n* (%)	23 (30.7)	104 (56.5)	120 (55.6)
Family history of CAD, *n* (%)	22 (29.3)	16 (8.8)	56 (26.7)
Killip class, *n* (%)			
I	64 (85.3)	140 (76.1)	173 (73.6)
II	7 (9.3)	28 (15.2)	32 (13.6)
III	2 (2.7)	5 (2.7)	21 (8.9)
IV	2 (2.7)	11 (6.0)	9 (3.8)
Beta-blockers, *n* (%)	66 (88.0)	142 (79.8)	157 (76.6)
ACE inhibitors, *n* (%)^a^	56 (74.7)	149 (83.7)	159 (76.4)
Primary PCI, *n* (%)	67 (89.3)	166 (90.2)	—
TIMI 3 flow, *n* (%)	71 (94.7)	174 (94.6)	—
Multivessel disease, *n* (%)	21 (28.0)	87 (47.3)	—
Median follow-up, days	1,490	562	730
Events, *n* (%)	28 (37.3)^b^	82 (44.6)^b^	77^c^

PCI, percutaneous coronary intervention.

^a^ ACE, angiotensin-converting enzyme.

^b^ Cohort study.

^c^ Nested case–control study.

**Table 2:** Patient characteristics according to the recurrence of acute coronary syndrome or cardiovascular death during the follow-up.

	Study 1			Study 2			REGICOR		
	No event (*n* = 47)	Event (*n* = 28)	*p*-value	No event (*n* = 102)	Event (*n* = 82)	*p*-value	No event (*n* = 160)	Event (*n* = 77)	*p*-value
Age	46.6 (6.5)	48.2 (5.2)	0.335	58.7 (13.9)	68.2 (12.7)	<0.001	61.7 (9.6)	62.3 (9.3)	0.623
Sex (male)	41 (87.2)	25 (89.3)	0.937	80 (78.4)	66 (80.5)	0.871	110 (68.8)	52 (67.5)	0.968
Smoking, *n* (%)	32 (68.1)	18 (60.7)	0.517	43 (42.2)	24 (29.3)	0.052	61 (38.6)	31 (40.3)	0.827
Hypertension, *n* (%)	15 (31.9)	13 (46.4)	0.270	56 (54.9)	57 (69.5)	0.065	89 (57.4)	49 (66.2)	0.259
Diabetes, *n* (%)	—	—	—	26 (25.5)	25 (30.5)	0.592	40 (27.0)	29 (40.3)	0.067
Dyslipidemia, *n* (%)	14 (29.8)	9 (32.1)	0.959	54 (52.9)	50 (61.0)	0.384	77 (53.8)	43 (58.9)	0.574
Family history of CAD, *n* (%)	17 (36.2)	5 (17.9)	0.172	13 (12.9)	3 (3.66)	0.045	33 (23.1)	23 (34.3)	0.040
Killip class, *n* (%)									
I	43 (91.5)	21 (75.0)	<0.001	84 (82.4)	56 (68.3)	<0.001	129 (81.1)	44 (57.9)	0.002
II	3 (6.4)	4 (14.3)		14 (13.7)	14 (17.1)		16 (10.1)	16 (21.1)	
III	1 (2.1)	1 (3.6)		3 (2.9)	2 (2.4)		9 (5.7)	12 (15.8)	
IV	0 (0)	2 (7.1)		1 (1)	10 (12.2)		5 (3.1)	4 (5.3)	
Beta-blockers, *n* (%)	41 (87.2)	25 (89.3)	0.995	82 (80.4)	60 (78.9)	0.548	118 (84.9)	38 (59.1)	<0.001
ACE inhibitors, *n* (%)	35 (74.4)	21 (75.0)	0.910	83 (81.4)	66 (86.8)	0.550	110 (78.0)	49 (73.1)	0.548
Standardized GRS	0.0 (1.02)	0.0 (0.99)	0.957	−0.11 (1.03)	0.13 (0.95)	0.078	−0.12 (0.99)	0.25 (0.97)	0.007

In the multivariable analyses, the effect size of the association between the standardized GRS and the risk of events was hazard ratio (HR) = 1.43 [95% confidence interval (CI): 0.92–2.22], HR = 1.16 (95% CI: 0.92–1.46), and OR = 1.31 (95% CI: 0.94–1.81), respectively.

Table 3 shows the predictive capacity of the clinical model, which included age, sex, Killip class, family history of CAD, and personal history of hypertension, along with the added predictive value of the GRS across studies. The inclusion of the GRS did not improve the discriminative capacity of the clinical model. In two studies, we observed a correct reclassification of the patients after including the GRS in the model.

**Table 3:** Predictive capacity of the clinical model and added predictive value of the genetic risk score.

	Study 1		Study 2		REGICOR		Meta-analysis	
	Estimate (95% confidence interval)	*p*-value	Estimate (95% confidence interval)	*p*-value	Estimate (95% confidence interval)	*p*-value	Estimate (95% confidence interval)	*p*-value
Clinical model C-statistic/AUC	0.633 (0.513 to 0.753)	0.030	0.678 (0.613 to 0.742)	<0.001	0.658 (0.576 to 0.739)	<0.001	—	
Clinical model + GRS C-statistic/AUC	0.669 (0.550 to 0.789)	0.005	0.684 (0.620 to 0.748)	<0.001	0.673 (0.593 to 0.754)	<0.001	—	
C-statistic/AUC change	0.036 (−0.028 to 0.101)	0.270	0.006 (−0.001 to 0.023)	0.526	0.016 (−0.023 to 0.055)	0.428	0.009 (−0.007 to 0.025)	0.248
Continuous NRI	0.511 (0.020 to 0.973)	0.040	0.051 (−0.237 to 0.360)	0.728	0.272 (−0.018 to 0.563)	0.066	0.293 (0.079 to 0.506)	0.007

NRI, net reclassification index.

### 3.2. Results of the meta-analysis

**Figure 1:**
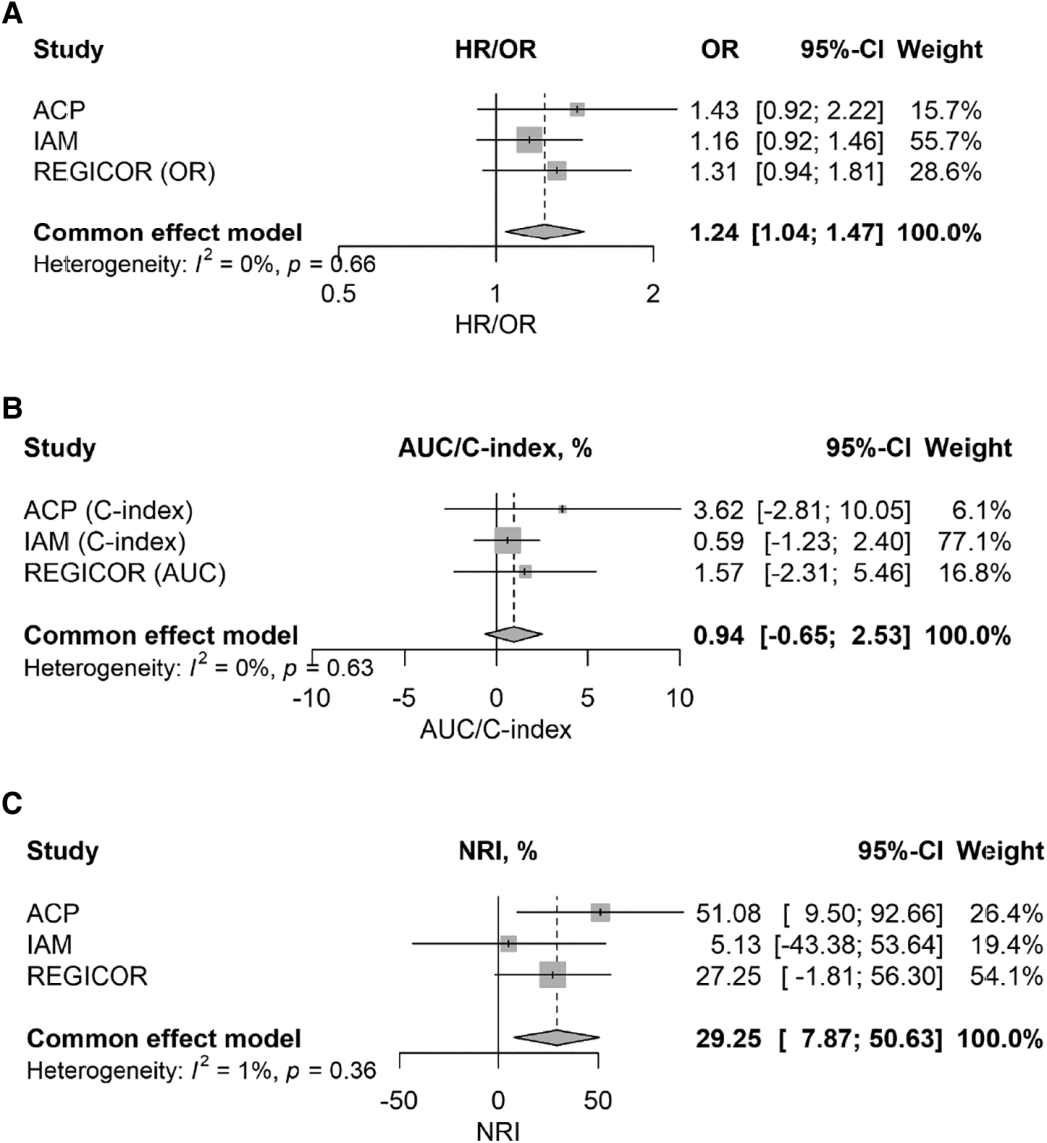
Forest plots of the meta-analysis showing the magnitude of the association between the standardized genetic risk score and risk of recurrences (**A**) and, with the addition of the genetic risk score in a basic clinical model, the changes in discriminative capacity (**B**) and reclassification (**C**) of the predictive model of recurrences.

Table 3 and Figure 1 show the results of the meta-analysis performed. A 24% increase in the risk of the outcomes of interest per standard deviation of the GRS was observed (95% CI: 4%–47%), without heterogeneity between studies (heterogeneity index −*I*^2^ = 0) (Figure 1A). The inclusion of the GRS did not increase the predictive capacity of the clinical model (change in C-statistic/AUC: 0.009; 95% CI: −0.007 to 0.025) (Figure 1B and Table 3) but improved its reclassification (continuous net reclassification index: 0.29; 95% CI: 0.08–0.51), without heterogeneity between studies (Figure 1C and Table 3).

## 4. Discussion

Our study highlights several important observations for considering GRS in clinical practice in the secondary prevention setting. First, we validated the effectiveness of a 12-SNP GRS in identifying patients with first myocardial infarction who are at the highest risk of recurrence and cardiovascular mortality. Second, adding this GRS to a clinical model improved its predictive capacity, since genetic risk information was independent and additive to all clinical variables currently used to assess subsequent cardiovascular risk. Third, we showed that this GRS, which was previously validated to assess cardiovascular risk in large healthy populations, can be useful in primary and secondary prevention.

The GRS for CAD, which was developed to assess the risk of a first cardiac event, has been extensively and positively evaluated (22). However, using GRS to predict recurrences among those with myocardial infarction has been less studied and with more variable results (8–15). In the present study, a GRS for CAD, independent of classical cardiovascular risk factors, was associated with a higher risk of recurrence in patients with a first myocardial infarction event, providing an enhanced risk assessment for those patients at risk of a secondary event. Moreover, the association between the standardized GRS and the risk of recurrences was very similar to that in a previous study we performed in a healthy Spanish population (HR = 1.24 vs. HR = 1.21) (6). Previous studies comparing the effect size of the association between a GRS and the risk of coronary events reported a lower magnitude of the association in patients with CAD than in individuals without the disease. This difference in the results could be related to two types of selection bias (14): either survival bias when selecting prevalent cases and exposure that could be related to a higher case-fatality or index event bias when stratifying on case status that could induce non-causal associations or attenuations between genetic variants and the index event. The consecutive and exhaustive inclusion of patients in the three studies included in this analysis might have prevented these types of bias.

The added predictive value of including a GRS in a predictive model for recurrences in patients with CAD has been less explored. Only one of the studies included in this analysis explored this hypothesis, showing an improvement in discrimination and reclassification—especially in patients with high low-density lipoprotein cholesterol (16). In this analysis, we confirmed the added value of the GRS in predicting coronary recurrences by improving the reclassification of patients according to their actual risk.

These findings could have clinical implications. First, the GRS identified a subgroup of patients with a higher risk of recurrences. Moreover, these patients could benefit from more intensive secondary preventive strategies. Previous studies suggested that patients with the highest burden of genetic risk obtained the largest relative and absolute clinical benefit from statin therapy (9) and, more recently, PCSK9 inhibitors (15, 23), providing evidence that contributes to the development of precision medicine in the cardiovascular domain.

One of the strengths of the study is the inclusion of consecutive patients and the meta-analysis of three studies with individual data. There are also some limitations that must be considered. First, the sample size of the studies is limited, but the results show high homogeneity and consistency across the studies. Second, the outcome is a composite of ACS recurrence and mortality; hence, individual outcomes could not be analyzed. Third, we cannot discard the survival bias related to the inclusion of patients who presented at the hospital and the exclusion of patients with out-of-hospital coronary death.

In conclusion, the GRS for CAD, independent of classical cardiovascular risk factors, was associated with an increased risk of cardiovascular recurrences in patients with first myocardial infarction.

## Data Availability

The original contributions presented in the study are included in the article/Supplementary material, further inquiries can be directed to the corresponding author.
