# Supplementary material for: Predictive capacity of a genetic risk score for coronary artery disease in assessing recurrences and cardiovascular mortality among patients with myocardial infarction
Source: Front Cardiovasc Med. 2023 Sep 14;10:1254066. doi: 10.3389/fcvm.2023.1254066 (PMC10537937; doi:10.3389/fcvm.2023.1254066)
Supplement: Supplementary file 1 [file Datasheet1.pdf]

Predictive capacity of a coronary artery disease genetic risk score for recurrences and cardiovascular mortality among patients with myocardial infarction.

Luis Miguel Rincón [1,2,3], Isaac Subirana [2,4], Candelas Pérez del Villar [1,2], Pedro L. Sánchez [1,2], José Luis Zamorano [2,3,5], Jaume Marrugat [2,4] and Roberto Elosua [2,4,6]

[1] Cardiology Department, Hospital Universitario de Salamanca – IBSAL, Universidad de Salamanca, Salamanca, Spain.
[2] Centro de Investigación Biomédica en Red de Enfermedades Cardiovasculares (CIBERCV), Spain.
[3] Universidad de Alcalá, Madrid, Spain
[4] Hospital del Mar Medical Research Institute, Barcelona, Spain
[5] Cardiology Department, Hospital Ramón y Cajal, Universidad de Alcalá, Madrid, Spain
[6] Department of Medicine, University of Vic-Central University of Catalonia, Vic, Spain

SUPPLEMENTARY DATA - METHODS

Supplementary Figure 1. Patient selection. Flowchart showing the inclusion/exclusion criteria for each cohort and meta-analysis selection process.

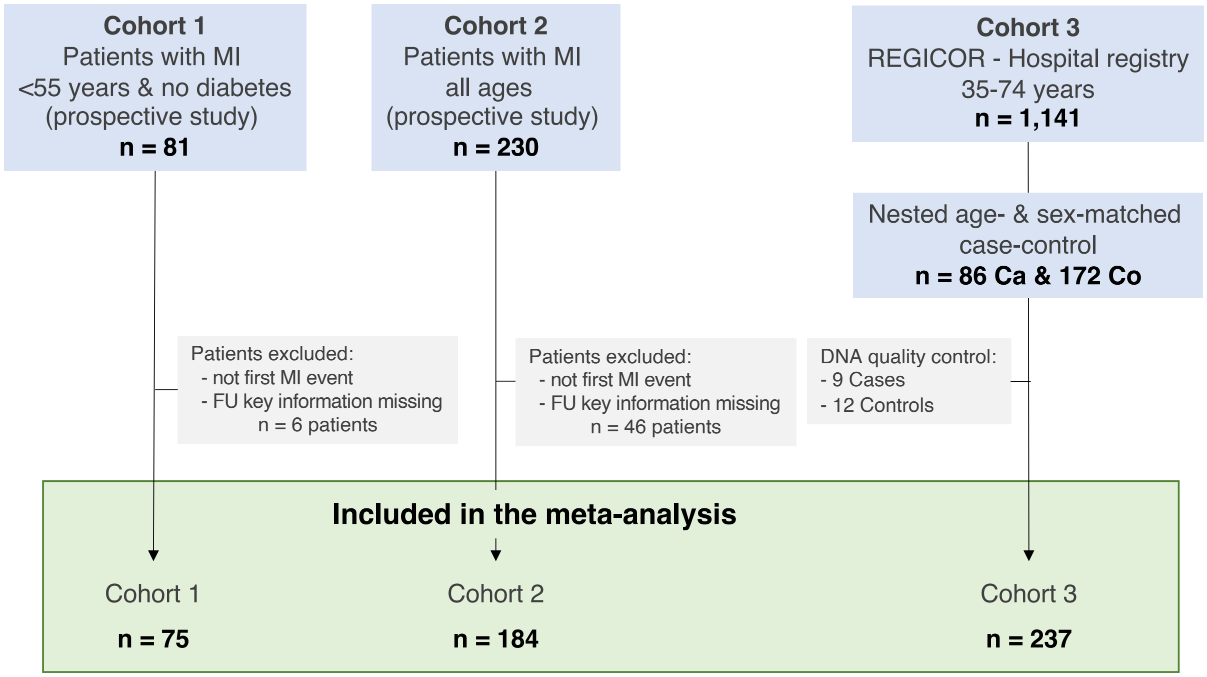

MI, myocardial infarction; FU, follow-up; REGICOR, *Registre Gironi del Cor* (Girona Heart Registry).
